# Intrinsic disorder in CYP1B1 and its implications in primary congenital glaucoma pathogenesis

**DOI:** 10.1007/s42485-025-00186-8

**Published:** 2025-05-13

**Authors:** Meghan Sharma, David Taylor Gonzalez, Michael Antonietti, Vladimir Uversky, Mak Djulbegovic

**Affiliations:** 1 John A. Moran Eye Center, University of Utah, Salt Lake City, UT, USA; 2 Hamilton Eye Institute, University of Tennessee Science Center, Memphis, TN, USA; 3 Bascom Palmer Eye Institute, University of Miami, 900 NW 17th Street, Miami, FL, USA; 4 Department of Molecular Medicine and, USF Health Byrd Alzheimer’s Research Institute, Morsani College of Medicine, University of South Florida, Tampa, FL, USA; 5 Wills Eye Hospital, Thomas Jefferson University, 840 Walnut Street, Philadelphia, PA 19107, USA

**Keywords:** AlphaFold, AlphaMissense, RIDAO, FuzDrop, D2P2, STRING

## Abstract

Cytochrome P450 1B1 (CYP1B1) plays a critical role in the pathogenesis of primary congenital glaucoma (PCG), a severe eye disorder that can lead to pediatric blindness if untreated. Increasing evidence suggests that intrinsically disordered proteins and regions (IDPs/IDPRs), which lack a stable three-dimensional structure, are significant in disease pathology due to their flexible nature, impacting protein interactions and function. This study explores the intrinsic disorder within CYP1B1 and its implications in the molecular mechanisms underlying PCG. We employed a comprehensive bioinformatics approach to assess the structural and functional properties of CYP1B1 using tools such as AlphaMissense, a tool crafted to evaluate the functional impact of missense mutations in proteins. Our structural analysis qualitatively demonstrated that CYP1B1 contains intrinsically disordered protein regions (i.e., spaghetti-like entities) that are structureless and flexible. Correlation analysis showed that disorder decreases exponentially relative to AlphaMissense predicted pathogenicity, with an exponential decay fit (*R*^2^ = 0.62), suggesting that highly disordered regions tend to harbor benign mutations. This study identifies critical intrinsically disordered regions within CYP1B1 and elucidates its complex interaction network, highlighting the potential role of these regions in PCG pathogenesis. The observed correlation between intrinsic disorder and reduced pathogenicity of mutations suggests that IDPRs may buffer against deleterious effects, providing a possible explanation for the variability in clinical outcomes associated with CYP1B1 mutations. These insights enhance our understanding of the molecular basis of PCG and offer potential targets for novel therapeutic interventions to combat this blinding childhood disorder.

## Introduction

Primary congenital glaucoma (PCG) is a genetic disease that causes abnormalities in the trabecular meshwork and anterior chamber angle, leading to elevated intraocular pressure (Faiq et al. 2015; Badawi et al. 2019; Thau et al. 2018). The prevalence of PCG varies by the population being studied and can range from 1 in 1250 in a subpopulation of Slovakia to 1 in 20,000 in Western countries (Shah et al. 2022). The disorder can be inherited through a sporadic or familial pattern. Approximately, 10–40% of all PCG cases are familial and are inherited in an autosomal recessive manner (Mocan et al. 2019).

There are three categories of PCG: neonatal onset (0–1 month), infantile onset (>1–24 months), and late onset (>24 months) (Thau et al. 2018). According to the Childhood Glaucoma Research Network, PCG is defined as the development of glaucoma in the absence of any acquired or nonacquired conditions with associated buphthalmos (Thau et al. 2018). Along with buphthalmos, other signs of PCG include epiphora, photophobia, blepharospasm, eye rubbing, and irritability. On examination, a clinician may observe elevated intraocular pressure (IOP), corneal edema, increased corneal diameter, corneal opacification with breaks in Descemet’s membrane called Haab striae, and enlarged axial length of the eye (Fang et al. 2015; Chouiter and Nadifi 2017). In addition, gonioscopy may show an iris that inserts anteriorly to the trabecular meshwork (Yu Chan et al. 2015). Such developmental abnormalities of the trabecular meshwork may cause IOP to increase due to obstruction of aqueous flow, leading to damage to the optic nerve and early vision loss (Yu Chan et al. 2015; Alayu et al. 2022).

Recent advances in technologies for PCG-associated gene identification have included linkage analysis, exome sequencing, whole-exome sequencing, and comparative genomic hybridization (Ling et al. 2020). Cytochrome P450 1B1 (CYP1B1) is the main gene implicated in PCG and is located at locus GLC3A (chromosome 2, 2p21) (Mocan et al. 2019; Khan et al. 2020). CYP1B1 is a member of the cytochrome P450 enzyme family and participates in the metabolism of substrates involved in development, including steroids and retinoids (Shah et al. 2022). It is believed that CYP1B1 variants in PCG lead to impaired metabolism of retinol, disrupting retinoid acid levels required for ocular development (Kumar et al. 2024). Studies have indicated that CYP1B1 plays a crucial role in regulating oxidative stress and maintaining the integrity of the trabecular meshwork and retinal tissues, with disruptions in its expression leading to increased oxidative stress, altered extracellular matrix production, and impaired cellular integrity and function in these ocular tissues (Muskhelishvili et al. 2001). Various mutations have been identified in the CYP1B1 gene leading to PCG with different founder mutations identified in different regions of the world: p.Gly61Glu in Iran, Lebanon, and Saudi Arabia, p.Arg390His in Pakistan, p. 4,339delG in Morocco, p.Glu387Lys in Europe, and p.Val320Leu in Vietnam and South Korea (Chouiter and Nadifi 2017). Other previously identified mutations include a c.107G>A missense mutation, a 12 base pair deletion mutation, and four homozygous CYP1B1 mutants (p.Ala288Pro, p.Asp242Ala, p.Arg355*, and p.Arg290Profs*37), for instance (Ling et al. 2020).

When compared to other genes involved in PCG, CYP1B1 mutations have been associated with more severe disease phenotypes as defined by increased mean IOP and a greater number of surgeries needed to treat (Faiq et al. 2015; Mocan et al. 2019; Khan et al. 2020). These alternative PCG genes may include gene CDT6/ANGPTL7 at loci GLC3B (chromosome 1, 1p36), an unknown gene at GLC3C (chromosome 14, 14q24), and LTBP2 at GLC3D (chromosome 14, 14q24) (Faiq et al. 2015; Mocan et al. 2019; Khan et al. 2020). It is likely that multiple genes contribute to PCG inheritance, as incomplete penetrance, variable expressivity, and non-dominant heterozygous CYP1B1 mutations have been observed (Garcia-Anton et al. 2017). The most widely accepted theory behind PCG’s pathophysiology is genetic changes lead to failure of the downstream CYP1B1 pathways, which leads to the development of abnormal ocular structures and promotes glaucomatous changes (Shah et al. 2022). CYP1B1 mutations specifically halt the migration of the embryonic neural crest cells destined to become trabecular meshwork, leading to the goniodysgenesis observed in PCG (Shah et al. 2022).

Genetic mutations in CYP1B1 can profoundly affect the phenotype of the human trabecular meshwork, impacting cellular function and gross anatomical structure. Our group is particularly interested in how these genetic mutations alter protein function, focusing on local protein structures. Specifically, we are interested in intrinsically disordered proteins (IDPs). IDPs and intrinsically disordered protein regions (IDPRs) are commonly found in proteomes of all living organisms and viruses (Dunker et al. 2000; Peng et al. 2015; Ward et al. 2004; Xue et al. 2012). These functional proteins or protein regions are characterized by the lack of stable 3D structure and possess highly flexible structures containing multiple functional segments (Uversky 2013; Uversky 2013; Daughdrill et al. 2005). Understanding the impact of mutations on these disordered regions can provide insights into disease mechanisms and potential therapeutic targets.

Our study aims to understand PCG in the context of the protein intrinsic disorder phenomenon, focusing on quantifying the intrinsically disordered protein regions (IDPRs) in CYP1B1, detailing the functional capabilities of these regions as compared to more structured regions, and characterizing its protein–protein interaction (PPI) network. We posit that CYP1B1 has extensive intrinsic structural disorder (i.e., IDPRs) and that genetic mutations previously identified by other groups impact the behavior of these regions, which leads to downstream development of PCT. If CYP1B1 gene mutations alter regions of intrinsic disorder within the protein, particularly depending on whether these mutations are localized in ordered or disordered regions, these molecular features may be considered as potentially viable targets for future therapies directed at PCG. Ultimately, this study seeks to contribute to the understanding of the molecular basis of disease for PCG.

## Methods

We performed a comprehensive structural bioinformatics analysis to elucidate previously unexplored molecular features of primary congenital glaucoma (PCG), focusing on the CYP1B1 protein. We utilized an approach to integrate various computational tools and databases. Structural assessments were carried out using advanced protein modeling techniques, while predictive tools provided insights into the pathogenicity of missense mutations. Amino acid composition and disorder predictions were analyzed using specialized profiling tools. Correlation analyses between intrinsic disorder and mutation pathogenicity were performed, and phase separation propensity and aggregation hot spots were evaluated using advanced prediction platforms. Disorder-related functionalities were further examined using comprehensive databases, and protein–protein interactions were mapped using curated interaction networks. Through this study, we aimed to reveal critical molecular insights into the role of CYP1B1 in PCG, shedding light on the molecular basis of the disease and potentially informing future therapeutic strategies.

### Protein sequences

The Universal Protein Resource (UniProt) (accessed on March 15, 2024, available at https://uniprot.org) serves as a repository for extensive protein data (UniPro 2019). For this search, the parameters included “gene: CYP1B1” and were filtered by “Human”. From the UniProt database, using the entry with the UniProt ID: Q16678, the standard amino acid sequence was obtained in a text-based FASTA format.

### Structural assessment

We accessed the AlphaFold2-generated protein structure of CYP1B1 from the AlphaFold Protein Structure Database (accessed on March 15, 2024, available at: https://alphafold.ebi.ac.uk). The goal of this portion of our analysis was to use AlphaFold, developed by Google’s DeepMind, which utilizes deep learning techniques to predict protein structural features with high accuracy as compared to the experiment. AlphaFold is renowned for its ability to generate highly accurate models of protein structures, making it an essential tool for understanding protein functionality and interactions (Varadi et al. 2022; Jumper et al. 2021). Although AlphaFold 3 was available at the time of this study, we opted not to use it since it was still in an early beta version (Abramson et al. 2024).

### AlphaMissense pathogenicity predictions

Building upon AlphaFold’s capabilities, DeepMind introduced AlphaMissense, a tool crafted to evaluate the functional impact of missense mutations in proteins, utilizing the structural insights offered by AlphaFold (Cheng et al. 2023). We employed AlphaMissense to evaluate the pathogenicity of missense mutations in CYP1B1, as missense mutations are the most common mutations seen in CYP1B1 according to ClinVar, a public archive of reports of human variations classified for diseases (Landrum et al. 2018). This deep learning model predicts the effects of amino acid substitutions on protein stability throughout the human proteome (Cheng et al. 2023). AlphaMissense assigns a pathogenicity score from 0 to 1, where scores closer to 1 indicate a higher likelihood of pathogenicity, and scores nearer to 0 suggest benign mutations. Scores between 0.34 and 0.564 are deemed less reliable and labeled “ambiguous” or of uncertain significance. We followed these cutoffs established by AlphaMissense, which were validated to achieve 90% precision on ClinVar data according to its initial publication, categorizing mutations as likely pathogenic, ambiguous, or likely benign. Typically, pathogenic variants adversely affect protein function and organismal fitness, while benign variants are likely to have minimal impact (Cheng et al. 2023).

To further analyze the structural implications of these pathogenicity predictions, we color coded the AlphaFold2-generated structure of CYP1B1 with the average AlphaMissense pathogenicity score for each residue, based on all possible mutations at that location. Residues were color coded on a gradient from blue (benign) to red (pathogenic), with white indicating ambiguous scores. This visualization highlights regions within the protein that may have higher or lower pathogenic potential, providing insights into the functional impact of missense mutations.

### Amino acid composition analysis

The CYP1B1 protein sequence underwent analysis through the Composition Profiler (accessed on March 15, 2024, available at: http://www.cprofiler.org/) (Vacic et al. 2007). This tool facilitated the examination of the enrichment or depletion of typical amino acids within CYP1B1 by comparing its amino acid profile against a background dataset consisting of proteins with high structural integrity, namely the PDB Select 25 (Vacic et al. 2007). Additionally, composition profiles for experimentally validated disordered proteins from the DisProt database and the distribution of amino acids in nature from the SwissProt database were generated for comparison (Sickmeier et al. 2007; Bairoch and Apweiler 2000). The assessment of amino acid enrichment or depletion is calculated using the formula (C_x_–C_order_)/C_order_, where C_x_ represents the concentration of a specific amino acid in the protein under investigation (i.e., CYP1B1) and C_order_ refers to its concentration in the proteins within the PDB Select 25.

### Prediction of disorder using commonly used predictors

To assess the role of intrinsic disorder in protein functionality, the analysis also aimed to identify disordered regions that may influence CYP1B1’s interaction with other biomolecules. Quantification of intrinsic protein disorder of the CYP1B1 sequence was performed using six widely recognized per-residue disorder prediction tools: PONDR^®^ VLXT (Romero et al. 2001), PONDR^®^ VL3 (Peng et al. 2005), PONDR^®^ VLS2 (Obradovic et al. 2005; Peng et al. 2006), PONDR^®^ FIT (Xue et al. 2010), and both IUPred2 (Short) and IUPred2 (Long) (Dosztányi et al. 2005 Dosztányi et al. 2005). A web crawler, accessed through the novel Rapid Intrinsic Disorder Analysis Online (RIDAO) tool (accessed on March 15, 2024, available at: https://www.ridao.app), was used to compile the predictions from these tools, offering an aggregated view of disorder ten-dencies in CYP1B1 by averaging the results (Dayhoff and Uversky 2022).

These predictors provide a disorder score for each residue, with a residue being considered disordered if it has a disorder score greater than or equal to 0.5. Using these values, we derived the percentage of predicted disordered residues (PPDR), which is the percentage of residues with a disorder score of 0.5 or higher. In these analyses, proteins were classified based on their PPDR, with two arbitrary cutoffs being used to classify proteins as highly ordered (PPDR < 10%), moderately disordered (10% ≤ PPDR < 30%), and highly disordered (PPDR ≥ 30%).

### Correlation analysis between disorder and pathogenicity

To investigate the relationship between intrinsic disorder and pathogenicity within the CYP1B1 protein, we conducted a correlation analysis between the mean disorder profile (MDP) and the AlphaMissense average pathogenicity score. The MDP was derived by averaging predictions from multiple disorder predictors, including PONDR^®^ VLXT, PONDR^®^ VL3, PONDR^®^ VSL2, PONDR^®^ FIT, IUPred2 (Short), and IUPred2 (Long).

We plotted the average pathogenicity scores against the MDP values to visualize potential trends and interactions. A moving mean with a window of ten residues was applied to the average pathogenicity scores to reduce noise and emphasize significant patterns. An exponential decay fit was incorporated to model the relationship.

Additionally, we color coded the pathogenicity scores: red for pathogenic, gray for ambiguous, and blue for benign mutations, providing a clear visual distinction of regions with different pathogenic potentials. To further elucidate these interactions along the protein sequence, we created a residue-wise plot of MDP and average pathogenicity scores. The MDP values were plotted, and the average pathogenicity scores were represented using the same color gradient as the scatter plot. This comprehensive visualization approach allowed us to identify and characterize regions within CYP1B1 where intrinsic disorder and mutation pathogenicity intersect, offering insights into the potential functional dynamics and disease associations of the protein.

### Assessment of phase separation propensity and aggregation hot spots in CYP1B1

Additional functional information on CYP1B1 was retrieved using the FuzDrop platform (accessed on May 27, 2024, available at: https://fuzdrop.bio.unipd.it/predictor) (Hardenberg et al. 2020). FuzDrop is a computational tool that assesses the likelihood of proteins undergoing liquid–liquid phase separation (LLPS) and provides information on the capability of a protein to undergo such phase separation (Horvath et al. 2022; Vendruscolo and Fuxreiter 2022). It identifies the presence and localization of droplet-promoting regions (DPRs), shows the location of aggregation hot spots (i.e., residues/regions that may promote the conversion of a protein to a liquid-like condensed state into a solid-like amyloid state), and provides the probability of a protein having residues/regions with cellular context dependence (i.e., those characterized by the ability of residues to switch between different binding modes) (Horvath et al. 2022).

FuzDrop classifies proteins with a high spontaneous LLPS tendency as ‘droplet-driving’ proteins, while those requiring additional interactions for droplet formation are considered ‘clients’. Proteins with an overall LLPS propensity score (pLLPS) of 0.60 or more are droplet drivers. Proteins with a lower pLLPS, but with specific droplet-promoting regions (DPRs), characterized by consecutive residues with a pLLPS of 0.60 or higher, are classified as droplet clients (Hardenberg et al. 2020).

### Assessment of disorder-related functionality using D_2_P_2_

The disorder-related functions within CYP1B1 were further characterized using the D2P2 database (accessed on May 27, 2024, available at: http://d2p2.pro/). (Oates et al. 2013) This resource integrates predictions from several algorithms including IUPred (Dosztányi et al. 2005), PONDR^®^ VLXT (Romero et al. 2001), PrDOS (Ishida and Kinoshita 2007), PONDR^®^ VSL2B (Obradovic et al. 2005; Peng et al. 2006), PV2 (Oates et al. 2013), and ESpritz (Walsh et al. 2012), to provide insights into the disorder characteristics of CYP1B1 and the consensus among these predictors. Additionally, it highlights functional SCOP domains (Andreeva et al. 2004; Murzin et al. 1995) as predicted by the SUPERFAMILY algorithm (Lima Morais et al. 2011) and includes data from the ANCHOR algorithm, which identifies binding sites expected to become structured upon interaction with their partners (Mészáros et al. 2009). The D_2_P_2_ database also suggests potential post-translational modifications as indicated by PhosphoSitePlus findings (Hornbeck et al. 2012).

### Protein–protein interaction network

To further evaluate the functional consequences of intrinsic disorder in CYP1B1, its amino acid sequence was analyzed using the Search Tool for the Retrieval of Interacting Genes (STRING) (accessed on March 15, 2024, available at: https://string-db.org/). STRING is a rigorously curated database that integrates both experimental and theoretical data to illustrate protein–protein interactions (Szklarczyk et al. 2019). The analysis focused on the canonical form of CYP1B1, specifying the interaction score threshold at the highest level of confidence (0.900) and setting the limit for displaying interactions to the maximum allowable number, which is 500.

## Results

### Protein sequences

We used the canonical amino acid sequence of the CYP1B1 protein in FASTA format for the bioinformatics analysis. The human CYP1B1 gene is 8.5 kilobases, containing three exons and two introns with an open reading frame starting at the 5’ end of the second exon. The open reading frame renders about 5.1 kilobases of mRNA (UniPro 2019). The CYP1B1 protein is the largest of the cytochrome P450 subfamilies with a predicted protein size of 543 amino acids (Alsubait et al. 2020).

### Structural assessment

**Fig. 1:**
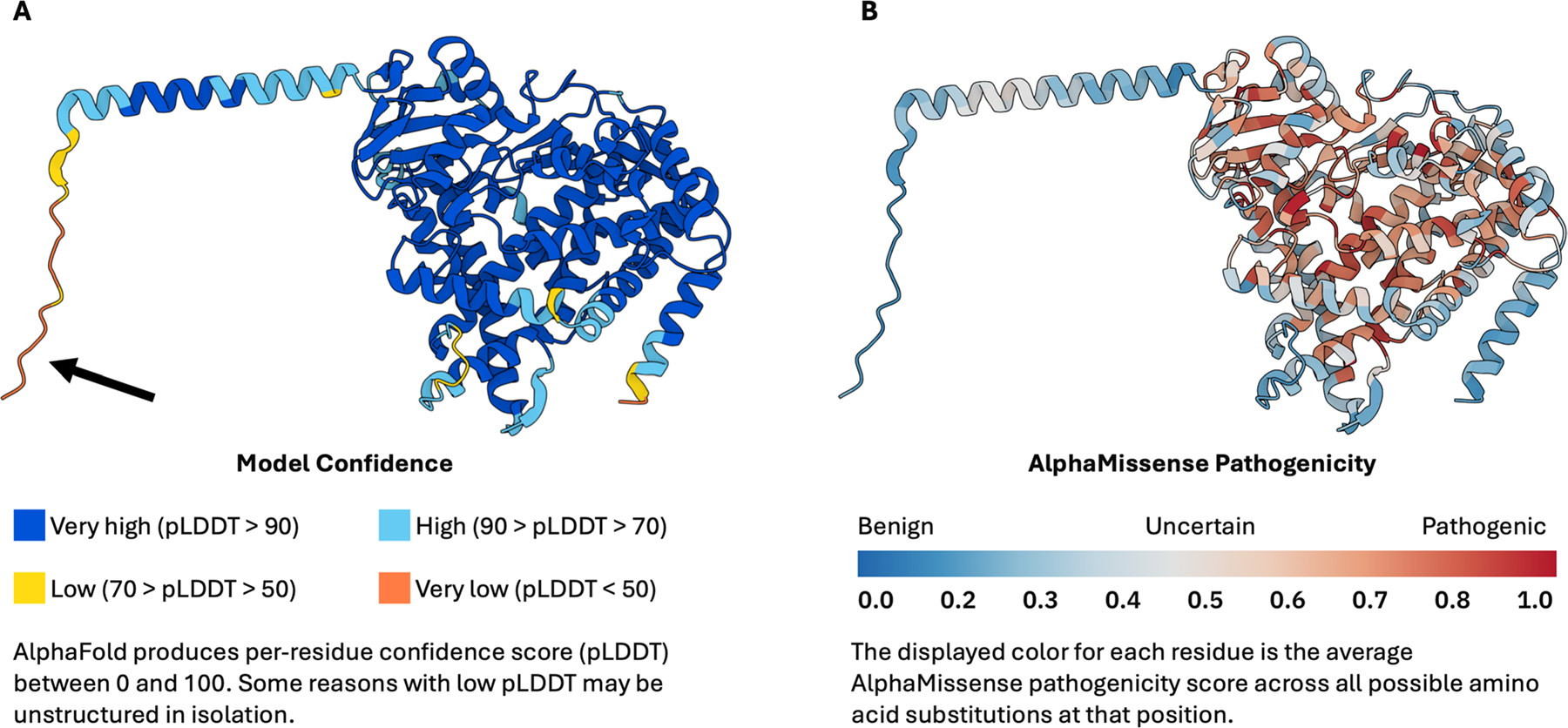
Visualization of disorder and pathogenicity through AlphaFold2-generated protein structures. **A** depicts the AlphaFold2-generated structure of CYP1B1. The model confidence in the structure is color coded by the predicted local distance difference test (pLDDT), ranging from blue (very high confidence) to red (very low confidence). Areas exhibiting greater model certainty are usually indicative of alpha-helices and beta-pleated sheets. Conversely, areas with reduced model confidence are likely to correspond to intrinsically disordered regions of proteins. The black arrow points to a location that might exhibit substantial disorder, lacking a clear structure. In **B**, residues are color coded based on their predicted pathogenicity from AlphaMissense, ranging from blue (benign) to red (pathogenic), with a color gradient through white at intermediate scores (ambiguous). The horizontal color reference bar on the right correlates the intensity of the color with the level of pathogenicity. The average pathogenicity score was determined to be 0.51 by AlphaMissense

AlphaFold2 predicted a 3D model of CYP1B1 that reveals a highly structured core and disordered peripheral areas, with string, spaghetti-like segments (Fig. 1A). Qualitative analysis of the 543 amino acids indicates that CYP1B1 includes flexible, unstructured regions known as intrinsically disordered protein regions (IDPRs). Structural portions of the protein are flanked by regions that lack definitive structure, which reside in residues at approximately 0–60, 140–180, 210–240, 280–320, and 520–543. The N- and C-terminal region of CYP1B1 (residues 1–12, 542–543) is also predicted to have low model confidence (i.e., high intrinsic disorder).

### AlphaMissense pathogenicity predictions

We then overlaid the AlphaMissense pathogenicity results onto the AlphaFold-generated structures. For each of the 543 amino acid residues in CYP1B1, we included all possible mutations and calculated an average pathogenicity score, which was used to color code the structure (Fig. 1B). On initial qualitative visual analysis, we observed that areas demarcated as low confidence by low pLDDT (disordered) were more likely to be benign according to AlphaMissense pathogenicity predictions. Further quantitative analysis was performed to refine our observations which will be discussed later in the study. It is important to note that these predictions do not indicate the probability or feasibility of these mutations occurring, but rather the likelihood that these mutations would cause functional detriments in the protein.

### Amino acid composition analysis

**Fig. 2:**
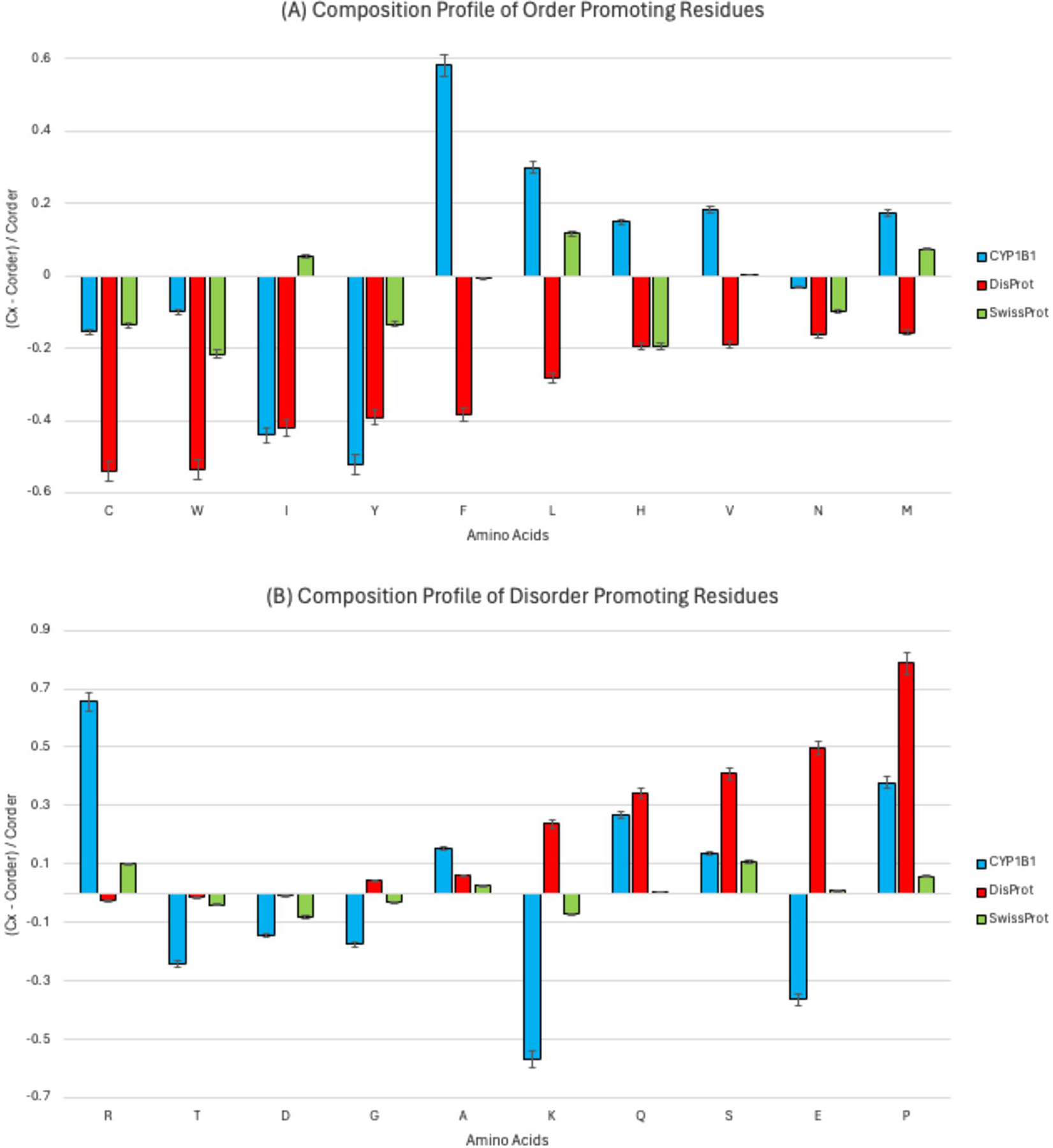
Amino acid composition profile of CYP1B1. The fractional difference is calculated as $\frac{C_{x-}C_{\text{order}}}{C_{\text{order}}}$, where *C*_*x*_ is the content of a given amino acid in the query set (CYP1B1), and *C*_*order*_ is the content of a given amino acid in the background set (Protein Databank Select 25). Additionally, composition profiles for experimentally validated disordered proteins from the DisProt database and the distribution of amino acids in nature from the SwissProt database were generated for comparison. The top panel (**A**) represents order-promoting residues, while the bottom panel (**B**) represents disorder-promoting residues. The amino acid residues are ranked from most order-promoting residues ((i.e., cysteine (C), tryptophan (W), isoleucine (I), tyrosine (Y), phenylalanine (F), leucine (L), histidine (H), valine (V), asparagine (N), and methionine (M)) to most disorder-promoting residues ((i.e., arginine (R), threonine (T), aspartate (D), glycine (G), alanine (A), lysine (K), glutamine (Q), serine (S), glutamate (E), and proline (P)). Positive values indicate enrichment, and negative values indicate depletion of amino acids. Amino acids marked with (*) are statistically significant for enrichment (F, L, H, V, and M) for the order-promoting residues. There was significant depletion of (C, W, I, Y, and N) (p value < 0.05) in the order-promoting residues as well. Amino acids marked with (*) are statistically significant for enrichment (R, A, Q, S, and P) for the disorder-promoting residues. There was significant depletion of (T, D, G, K, and E) (p value < 0.05) in the disorder-promoting residues as well. Statistical significance for enrichment or depletion was calculated using a two-sample t test, with a Bonferroni correction for multiple comparisons (p < 0.05)

The composition of the CYP1B1 sequence was analyzed and compared through visualization with the experimentally validated protein composition profile from the DisProt database and the natural distribution of amino acids from the SwissProt database (Fig. 2). Figure 2A shows the composition profile of order-promoting residues, and Fig. 2B depicts the composition profile of disorder-promoting residues. In both plots, the amino acids are ranked from left to right, from the most order promoting to the most disorder promoting.

Amino acids that appear more frequently than expected were assigned positive values, signifying enrichment, while those less frequent received negative values, indicating depletion. In our findings, residues such as phenylalanine (F), leucine (L), histidine (H), valine (V), and methionine (M) were significantly enriched, and cysteine (C), tryptophan (W), isoleucine (I), tyrosine (Y), and asparagine (N) were significantly depleted among order-promoting amino acids (p value < 0.05). For disorder-promoting residues, arginine (R), alanine (A), glutamine (Q), serine (S), and proline (P) were significantly enriched, whereas threonine (T), aspartate (D), glycine (G), lysine (K), and glutamate (E) were significantly depleted (p value < 0.05).

### Prediction of disorder using commonly used predictors

**Fig. 3:**
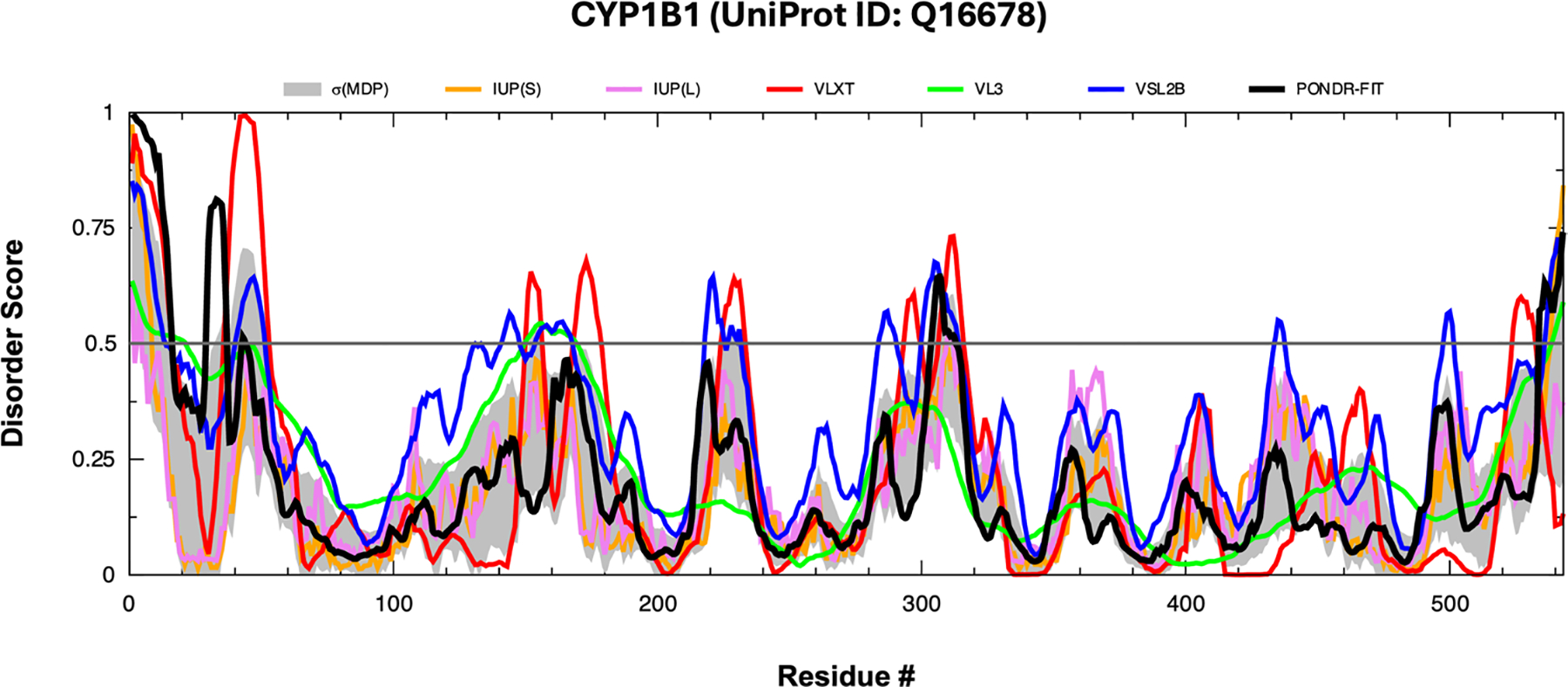
Evaluation of the intrinsic disorder predisposition of CYP1B1. Intrinsic disorder profiles for each residue compiled by RIDAO that integrate results from multiple predictors, including PONDR^®^ VLXT, PONDR^®^ VL3, PONDR^®^ VSL2, PONDR^®^ FIT, IUPred_long, and IUPred_short. The protein’s mean disorder profile (MDP) is determined by averaging the disorder scores from these individual predictors. A light shaded area represents the error distribution of the MDP. A thin black line marks the 0.5 disorder score, serving as the boundary between ordered and disordered states; residues or regions scoring above 0.5 are considered disordered, whereas those scoring below 0.5 are deemed ordered

Following the assessment of CYP1B1’s amino acid makeup, we aimed to evaluate the intrinsic disorder of each residue. Utilizing algorithms such as PONDR^®^ VLXT (Romero et al. 2001), VL3, VSL2, FIT, and both IUPred-Long and IUPred-Short, we calculated the disorder scores for each amino acid in CYP1B1 (Fig. 3) (Dayhoff and Uversky 2022) (Vacic et al. 2007). CYP1B1 was forecasted to be moderately disordered by PONDR^®^ VLXT at 15.65% and VSL2 at 17.86%, while PONDR^®^ VL3 and FIT, along with IUPred-Long and IUPred-Short, indicated it as highly ordered, with PPDRs of 8.47%, 8.29%, 1.10%, and 2.39%, respectively.

### Correlation analysis between disorder and pathogenicity

In our study, we conducted a detailed correlation analysis between the mean disorder profile (MDP) and the AlphaMissense average pathogenicity score within the CYP1B1 protein. The MDP was derived by averaging predictions from multiple disorder predictors.

**Fig. 4:**
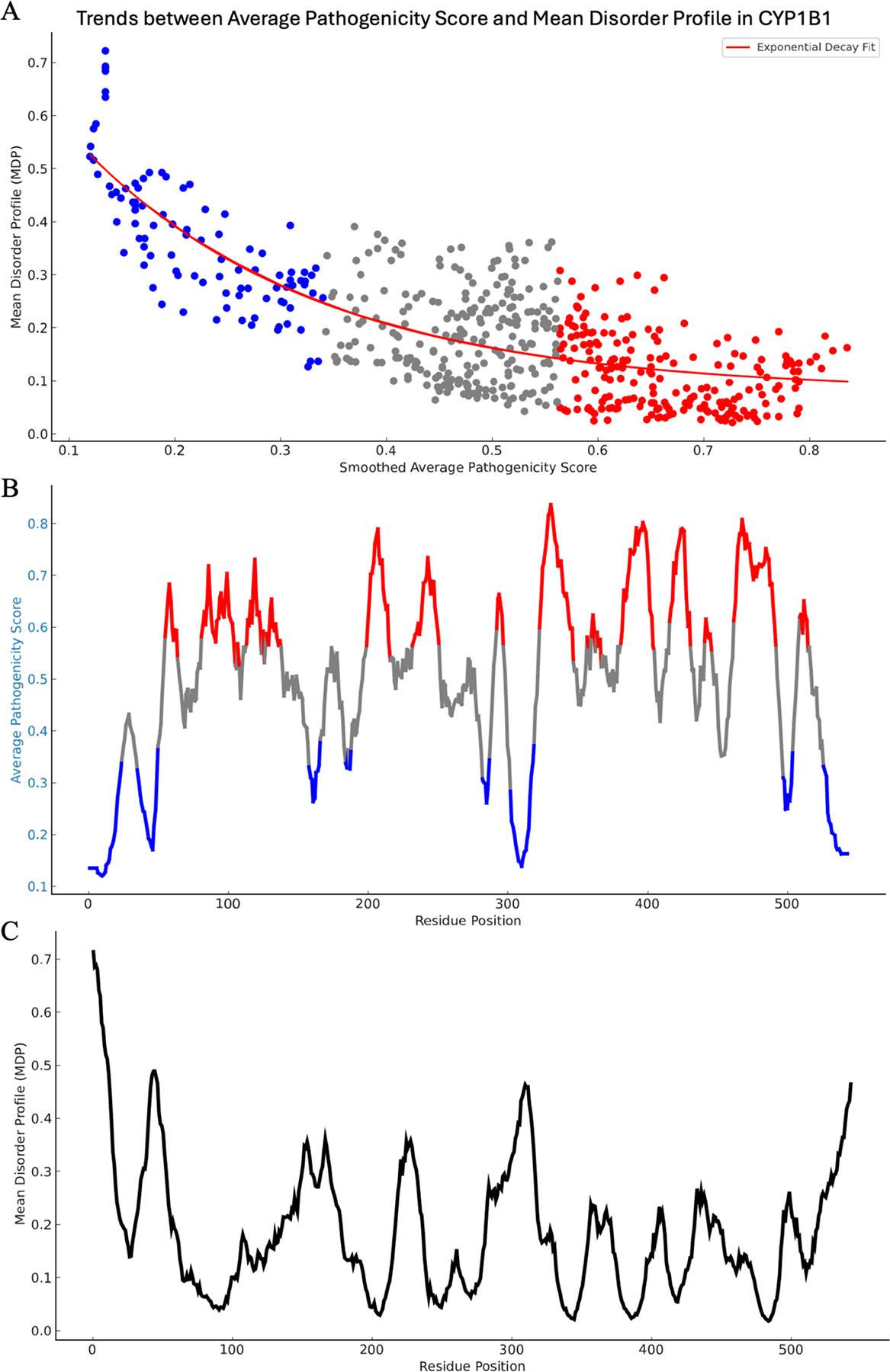
Correlation between mean disorder profile (MDP) and AlphaMissense average pathogenicity score in CYP1B1. This figure illustrates the relationship between the mean disorder profile (MDP) and the AlphaMissense average pathogenicity score for CYP1B1, highlighting regions of interest within the protein. **A** presents a scatter plot of smoothed average pathogenicity scores against MDP values, with an exponential decay fit shown in red, and each dot represents an amino acid residue. A moving mean with a window of ten residues was applied to reduce noise and clarify trends. A color gradient was used to highlight pathogenic (red), ambiguous (gray), and benign (blue) mutations based on the predicted pathogenicity scores. The R^2^ value of 0.62 indicates the proportion of variance in MDP explained by the average pathogenicity score. **B** depicts the MDP and **C** the average pathogenicity scores along the residue positions of CYP1B1. The average pathogenicity score is shown as a color gradient, with blue indicating benign, gray indicating ambiguous, and red indicating pathogenic mutations. The MDP is displayed in black. This plot integrates multiple disorder predictors, including PONDR^®^ VLXT, PONDR^®^ VL3, PONDR^®^ VSL2, PONDR^®^ FIT, IUPred_long, and IUPred_short, averaged to form the MDP

In Fig. 4A, we present a scatter plot of the smoothed average pathogenicity scores against MDP values for individual amino acids. The application of a moving mean with a window of ten residues reduced noise and clarified trends. An exponential decay fit, shown in red, was incorporated into the plot, aligning well with our data. This fit indicated a moderate–strong exponential decay fit with an R^2^ value of 0.62, suggesting that disorder decreases exponentially relative to predicted pathogenicity. This adjusted R^2^ for the exponential decay fit was calculated as $R^{2}=1-\frac{\sum_{i=1}^{n}{\left(y_{i}-\hat{y}\right)}^{2}}{\sum_{i=1}^{n}{\left(y_{i}-\bar{y}\right)}^{2}}$. In other words, regions with higher disorder tend to have lower pathogenicity scores, implying that the most disordered regions are more likely to be predicted as benign mutations by AlphaMissense.

Figure 4B, C provides a residue-wise visualization of the MDP and average pathogenicity scores along the length of the CYP1B1 protein. In these figures, residues are color coded based on their pathogenicity scores, with blue indicating benign, gray indicating ambiguous, and red indicating pathogenic mutations. The MDP values are plotted in black. This side-by-side visualization reveals that peaks of high disorder align closely with areas of low pathogenicity, and conversely, regions with lower disorder often correspond to higher pathogenicity scores. This comprehensive approach allowed us to identify and characterize regions within CYP1B1 where intrinsic disorder and mutation pathogenicity intersect, offering insights into the potential functional dynamics and disease associations of the protein.

### Assessment of phase separation propensity and aggregation hot spots in CYP1B1

**Fig. 5:**
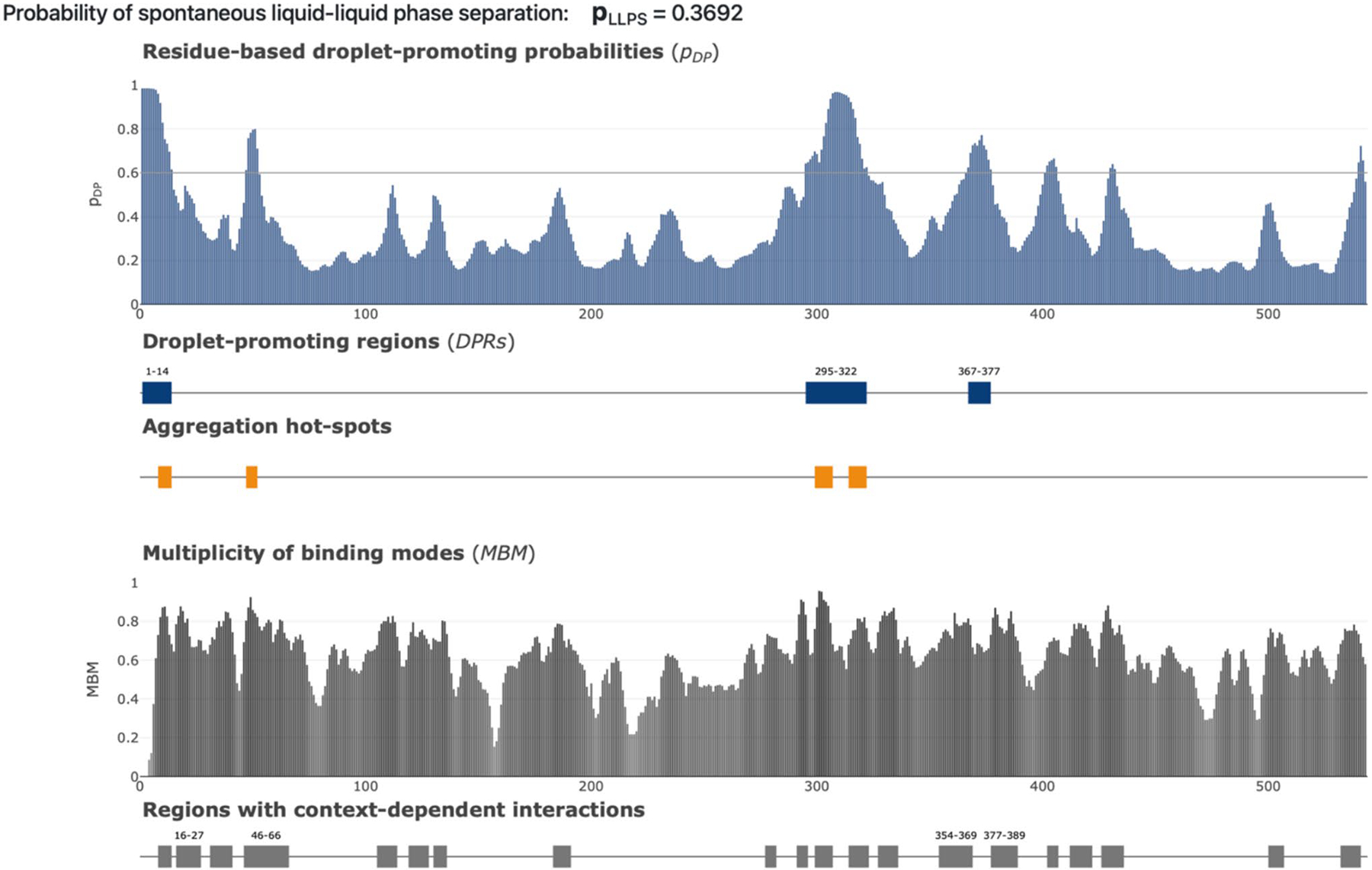
Evaluation of liquid–liquid phase separation propensity of human CYP1B1 by FuzDrop. We assessed the propensity of human CYP1B1 to undergo liquid–liquid phase separation (LLPS) using the FuzDrop computational tool. The overall probability of spontaneous LLPS for CYP1B1 is indicated as 0.3692, suggesting a moderate likelihood of promoting droplet formation. The top panel displays the residue-based droplet-promoting probabilities (p_DP_) along the CYP1B1 sequence, with higher values indicating regions more likely to promote droplet formation. Below this, the blue bars represent droplet-promoting regions (DPRs), highlighting specific segments within the protein that have a higher propensity for LLPS. The orange bars indicate aggregation hot spots, which are regions that could facilitate the transition from a liquid-like to a solid-like amyloid state, potentially leading to protein aggregation. The bottom panel illustrates the multiplicity of binding modes (MBM), which indicates regions with context-dependent interactions that can switch between disordered and ordered binding states. The plot underneath shows these regions with context-dependent interactions, marked in gray, with specific highlighted segments denoting areas of interest

Using the FuzDrop platform, we identified several key characteristics of the CYP1B1 protein related to phase separation propensity and aggregation hot spots. Figure 5 indicates that CYP1B1, with a pLLPS of 0.3692, has a low propensity to phase separate on its own, but includes three DPRs at residues 1–14, 295–322, and 367–377. Thus, CYP1B1 is identified as a droplet client, with its DPRs coinciding with extensive internal IDPRs, particularly at residues 1–14 and 295–322.

FuzDrop identifies aggregation hot spots, which are specific residues or regions that may facilitate the transition from a liquid-like to a solid-like amyloid state by engaging in both structured and unstructured binding, potentially triggering the aggregation process (Horvath et al. 2022; Vendruscolo and Fuxreiter 2022). Figure 5 highlights that human CYP1B1 possesses four such hot spots (residues 8–14, 47–52, 299–307, 314–322)

Figure 5 indicates that CYP1B1 features 20 such context-dependent regions, with the largest of these located at residues 16–27, 46–66, 354–369, and 377–389. Notably, these regions either coincide with, are encompassed by, or are closely adjacent to the intrinsically disordered protein regions (IDPRs) identified in CYP1B1.

### Assessment of disorder-related functionality using D_2_P_2_

To explore the disorder-related functions within CYP1B1, we utilized the D_2_P_2_ database. The D_2_P_2_ platform quantifies the functional intrinsic disorder of each protein and assesses its relationship with post-translational modifications (PTMs) and disorder-based binding potential. The D_2_P_2_ output for CYP1B1 demonstrates relative agreement among the disorder-based predictors, indicating regions of intrinsic disorder throughout the protein (Fig. 5).

CYP1B1 is characterized by one superfamily domain, Cytochrome P450, spanning residues 51–523, with further confirmation from the Pfam database indicating the Cytochrome P450 domain from residues 51–521.

Our analysis revealed no molecular recognition features (MoRFs) or predicted PTMs within the CYP1B1 sequence, suggesting a lack of specific post-translational modifications influencing disorder-based interactions. In addition, the most disordered regions in CYP1B1 are primarily located at the N-terminus, with many predictors showing agreement within the predicted domains. These disordered regions coincide with the functional Cytochrome P450 domain, suggesting a potential interplay between intrinsic disorder and protein function. The disorder identified here is consistent with our results from the RIDAO per-residue disorder predictions and the quantitative and qualitative AlphaFold structure predictions.

### Protein–protein interaction network

**Fig. 6:**
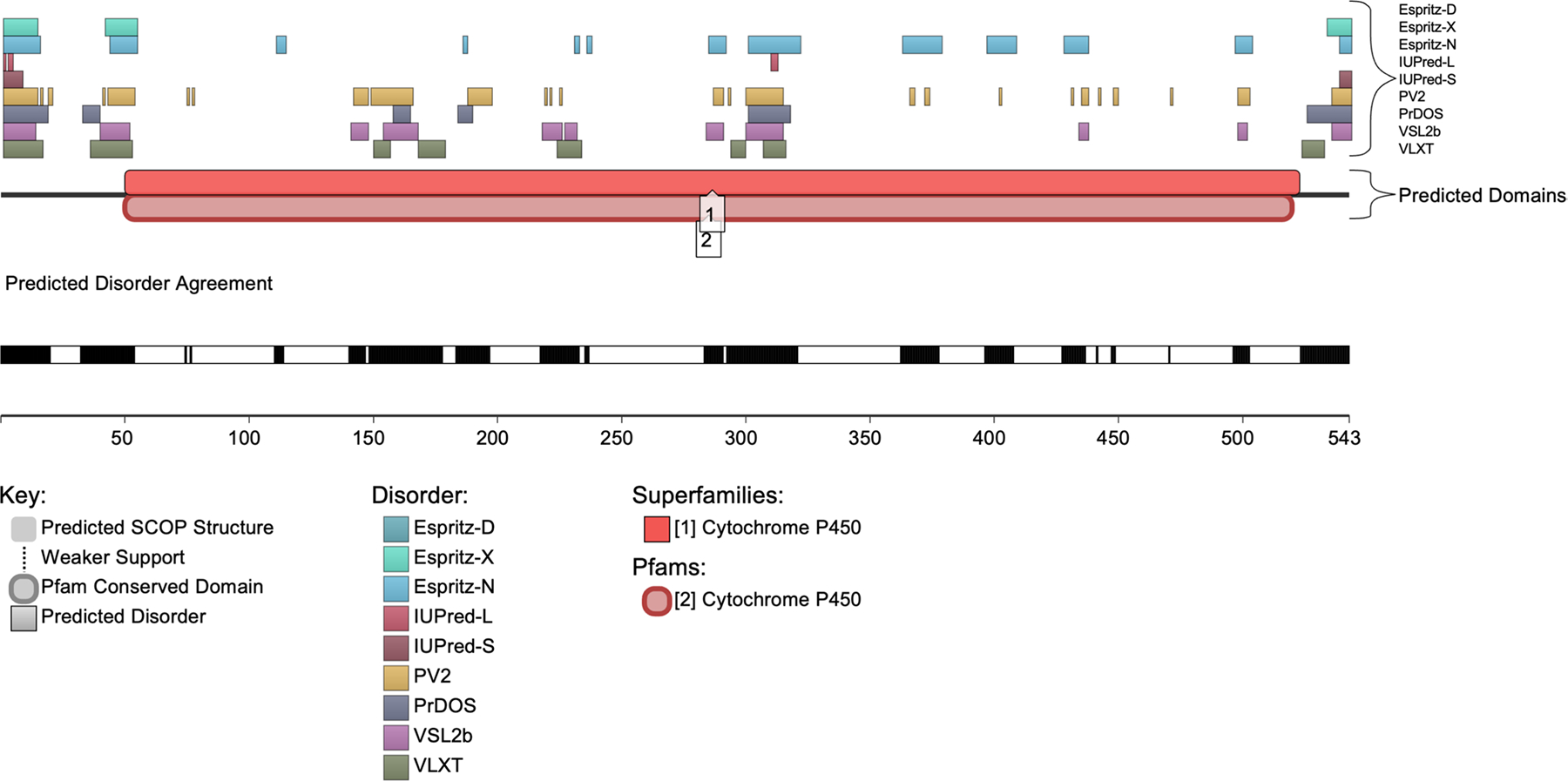
Visualization of predicted intrinsic disorder and functional domains in CYP1B1 using the D2P2 Database. This figure presents the predicted intrinsic disorder and functional domain structure of the CYP1B1 protein. The top section shows regions of disorder as predicted by various algorithms, including Espritz-D, Espritz-X, Espritz-N, IUPred-L, IUPred-S, PV2, PrDOS, VSL2b, and VLXT, each labeled accordingly. The middle section displays the predicted domain structures, highlighting the Cytochrome P450 superfamily domain and Pfam conserved domains within CYP1B1. The predicted SCOP structures are shown in gray, with lighter shading indicating areas of weaker support. Below this, the disorder agreement section illustrates the consensus on predicted disorder across the multiple prediction tools, represented by the black and white bar. Black segments indicate regions of higher predicted disorder. Notably, the analysis revealed no molecular recognition features (MoRFs) or post-translational modifications (PTMs) within the CYP1B1 sequence

**Fig. 7:**
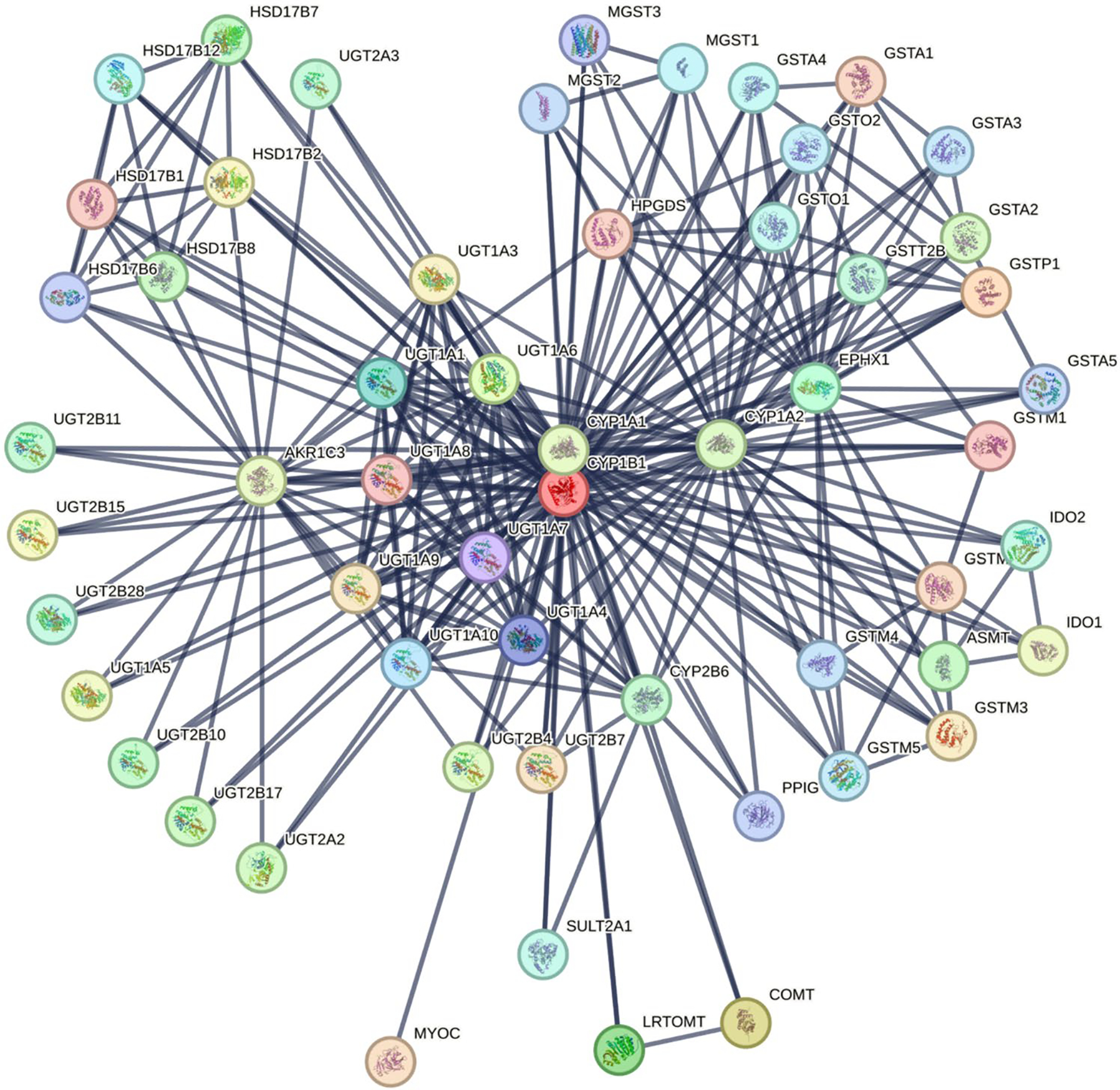
Protein–protein interaction network of cytochrome P450 1B1 (CYP1B1) revealed by the Search Tool for the Retrieval of Interacting Genes (STRING). A detailed STRING analysis, set at the highest confidence level of 0.900, focuses on the canonical form of CYP1B1. This analysis showcases a dense interaction network, with line thickness indicating the strength of data support. The network includes 56 nodes and 268 edges, demonstrating robust connectivity (average node degree: 9.57) and an average local clustering coefficient of 0.892, indicating a tightly interconnected cluster of proteins. The limit for displaying interactions was set to the maximum allowable number of 500. The observed connectivity significantly exceeds the expected 60 edges, and with a protein–protein interaction (PPI) enrichment p value of less than 1.0e–16

The final phase of our bioinformatics analysis allowed us to visualize CYP1B1’s extensive binding partners using STRING (Fig. 6). A detailed STRING analysis with high confidence (0.900) uncovered a complex network indicating CYP1B1’s key role in primary congenital glaucoma development. The network comprises 56 nodes and 268 edges, with an average node degree of 9.57, indicating a higher degree of connectivity than expected. This network, with a high average local clustering coefficient of 0.892, forms a tight protein cluster. The notable connectivity, exceeding the predicted 60 edges, coupled with a protein–protein interaction (PPI) enrichment p value of <1.0e-16, underscores the significance of CYP1B1’s interactions in the cellular context and its implications in ocular development and pathologies. Figure 7.

## Discussion

Intrinsic disorder is a phenomenon that has been studied in several diseases of the human eye, including uveal melanoma, conjunctival melanoma, and ocular surface squamous neoplasm (Djulbegovic et al. 2022; Djulbegovic et al. 2022; Djulbegovic et al. 2021). The present study is the first to evaluate the role of IDPRs in glaucoma pathogenesis, particularly primary congenital glaucoma. In our comprehensive study, we utilized a bioinformatics approach to elucidate the structural and functional nuances of the CYP1B1 protein.

Qualitative assessment of the CYP1B1 AlphaFold structure revealed a structured core integrated with many intrinsically disordered regions (IDPRs), which, when integrated with AlphaMissense predictions, indicated that regions of high disorder generally exhibited lower pathogenic potential. This relationship was quantitatively supported by an exponential decay fit (R^2^ = 0.62), demonstrating that disorder decreases exponentially relative to predicted pathogenicity, aligning with findings from the AlphaMissense publication that suggested similar trends across the human proteome (Cheng et al. 2023).

Our amino acid composition analysis highlighted that CYP1B1 is enriched with both order-promoting and disorder-promoting residues, further suggesting a complex structural environment. Per-residue disorder predictors classified the protein to be moderately disordered (PONDR^®^ VLXT and VSL2) or highly ordered (PONDR^®^ VL3, FIT, IUPred-Long, and IUPred-Short). FuzDrop predictions revealed key functional aspects of the protein, identifying three regions that promote droplet formation and four aggregation-prone hot spots. Furthermore, the FuzDrop analysis highlighted 20 context-dependent regions within the protein, which demonstrate a capacity to transition between various binding states, illustrating its complex interactions. The large protein–protein interaction network predicted by STRING emphasizes CYP1B1’s significant biological interconnectedness and its role in many pathways that, when disrupted by mutations may lead to pathogenesis. By mapping these structural insights against known pathogenic mutations, our study provides a clearer picture of how intrinsically disordered regions within CYP1B1 might influence its function and stability, offering potential targets for therapeutic intervention in PCG.

The most common mutation found is p.Gly61Glu, which has been identified in Iran, Portugal, Saudia Arabia, and more recently in Brazil and Vietnam (Chouiter and Nadifi 2017; Haddad et al. 2021). According to our RIDAO analysis, residue 61 is in an ordered region. Both glycine and glutamate are considered disorder-promoting residues. This mutation from a disordered residue (Gly) to a more disordered residue (Glu) in an order-promoting region may contribute to pathogenesis. Moreover, in our AlphaMissense analysis, we demonstrated that mutations located in ordered regions of CYP1B1 were more likely to be pathogenic than mutations found in disordered regions. This may suggest that any mutations in ordered regions might have a disproportionate impact on protein function, including significant changes to post-translational modifications or transient interaction sites. The pathogenicity score for p.Gly61Glu from AlphaMissense was predicted to be 0.9839, suggesting high pathogenicity and substantiating this hypothesis.

Several other mutations have been identified as founder mutations in specific regions of the world. The founding mutation in Europe is p.Glu387Lys, which is in an extremely ordered region at residue 387 (the VLXT model predicted a disorder score of close to zero around residue 390) (Chouiter and Nadifi 2017). Similar to the previous mutation, the change in p.Glu387Lys is from a disorder-promoting residue to the most disorder-promoting residue and is likely to cause pathogenic change. This was supported by a high AlphaMissense pathogenicity score of 0.9508. The founder mutation in Pakistan, p.Arg390His, is seen as a missense mutation in the same highly ordered region as the previous mutation at residue 390 (Chouiter and Nadifi 2017). While this change from a disorder-promoting residue to an order-promoting residue is not what would be expected for pathogenicity, perhaps any change in an extremely ordered region, especially at residue 390, could lead to pathogenicity. This theory is confirmed by an AlphaMissense pathogenicity score of 0.8207 characterizing the mutation as pathogenic. Nevertheless, this pathogenicity score is lower than the scores seen for the previous two mutations that favored disorder-promoting residues. Therefore, this suggests that the most pathogenic mutations are those that favor disorder-promoting residues in ordered regions followed by order-promoting mutations in ordered regions. Finally, in Vietnam and South Korea, the p.Val320Leu founder mutation is seen as a change from an order-promoting residue to a residue that is more order-promoting and is a unique deviation from the previous mutations (Chouiter and Nadifi 2017). This p.Val320Leu mutation, however, is in a more disordered region of the protein than the mutations previously discussed. While disordered regions were shown to be less pathogenic than ordered regions from our AlphaMissense analysis, this mutation suggests that residue changes that favor order in disorder-promoting regions may also contribute to pathogenicity. Although this may be possible, this mutation was predicted to be ambiguous at 0.381 by AlphaMissense, indicating that mutations in ordered regions are still likely to be more pathogenic than mutations in disordered regions. We focus on missense mutations in this paper; however, it is important to note that other mutations may also be seen in CYP1B1, such as frameshift mutations, which can have a profound deleterious effect on a protein’s function as it can truncate the protein significantly (Haddad et al. 2021).

This is also one of the first studies to predict the pathogenicity of the missense mutations seen in CYP1B1. AlphaMissense predicted ordered regions to be more pathogenic. As outlined in the preceding paragraphs, many CYP1B1 mutations may be explained through this idea. However, while our proteomics analysis demonstrates how the intrinsic structure of the CYP1B1 protein may exhibit pathogenicity, further research must be pursued to understand the extent to which these intrinsically disordered regions contribute to PCG development. Moreover, there are several other limitations to this study. While we performed a proteomics analysis using several tools, the databases and tools used in this study cannot provide a comprehensive representative of CYP1B1-related proteomics. This study is also a bioinformatics-based investigation using predictive tools, and clinical outcomes and pathogenicity predictions are not exhaustive from the predictive models we used in this study, such as AlphaMissense. Additional basic science and clinical research would be necessary to determine the exact role of IDPRs in CYP1B1 and novel treatment options for PCG.

As we contemplate future research directions, gene therapy presents an intriguing possibility for addressing PCG, particularly through the lens of structural biology. Several studies have explored the use of gene therapy in glaucoma. Animal studies have shown gene therapy may have effective measures on aqueous humor and could exert optic ganglion cell protection (Ling et al. 2020). Currently, however, there are no gene therapies for PCG, as gene therapy for the treatment of ocular disease has typically focused on retinal and optic nerve degenerations (Sulak et al. 2024). The CYP1B1 protein, central to our study, features a complex interplay of structured cores and IDPRs, which are pivotal for its function and regulation. Such disordered regions could provide novel targets for gene therapy, aimed at modulating these structural domains to correct dysfunctional interactions and mutations identified in PCG. Although current treatment strategies focus predominantly on surgical interventions and pharmacological management to control intraocular pressure, the detailed understanding of CYP1B1’s structure opens the potential for developing targeted gene therapies (Mocan et al. 2019). These therapies could specifically address the dynamic structural components of CYP1B1, targeting disordered regions implicated in the disease pathogenesis. By leveraging the insights from our proteomic analyses, future therapeutic approaches could be designed to stabilize these regions, potentially preventing or correcting the protein misfolding and dysfunction associated with PCG. This approach may enhance the specificity and efficacy of treatment and also aligns with the ongoing advances in molecular therapies for ocular diseases.

## Limitations

Our findings highlight the correlation between intrinsic disorder and mutation pathogenicity, but the underlying molecular mechanisms remain to be fully elucidated. Future studies could build on this work by integrating advanced structural modeling and experimental validation to uncover these mechanistic effects.

Additionally, the AlphaFold2 (AF2) model used in this study was selected for qualitative exploration and visualization purposes, rather than quantitative analysis of conformational diversity or molecular interactions. While AlphaFold3 (AF3) offers improved accuracy and the ability to simulate interactions between DNA, RNA, and proteins, its beta-phase status at the time of this study and our focus on single-protein analysis made AF2 the appropriate choice. We did not evaluate multiple AF2 models, use structural templates, or explore alternative sequence alignments, as these were outside the scope of this study. Future research could consider these approaches and leverage AF3 for deeper insights into conformational variability, protein dynamics, and multi-molecule interactions.

Lastly, while we employed a set of five well-established disorder predictors validated for analyzing CYP1B1, we acknowledge that incorporating newer high-performance predictors from recent CAID assessments could enhance the robustness of our findings. Future studies should aim to integrate these tools for a more comprehensive analysis.

## Conclusion

Our research demonstrates a complex interplay between the ordered and disordered regions of the CYP1B1 protein, which is crucial for understanding its multifaceted role in primary congenital glaucoma (PCG). While CYP1B1 is predominantly an ordered protein, its strategically positioned disordered regions appear to enhance its functional flexibility, allowing it to engage in a diverse range of protein–protein interactions. This structural arrangement supports CYP1B1’s involvement in various cellular functions and also correlates with its extensive interaction network, underscoring its adaptability and diverse role in biological processes. These insights suggest that the disordered regions of CYP1B1 could be key targets for therapeutic interventions aimed at modulating its activity and mitigating the pathological effects of genetic mutations associated with PCG.

## Data Availability

No datasets were generated or analysed during the current study.
